# Supplementary material for: Reduced Resting-State Connectivity in the Precuneus is correlated with Apathy in Patients with Schizophrenia
Source: Sci Rep. 2020 Feb 13;10:2616. doi: 10.1038/s41598-020-59393-6 (PMC7018974; doi:10.1038/s41598-020-59393-6)
Supplement: Supplementary file 1 — SupplementaryMethods_Figure. [file 41598_2020_59393_MOESM1_ESM.pdf]

Reduced Resting-State Connectivity in the Precuneus is correlated with Apathy in Patients with Schizophrenia

Caroline Garcia Forlim [a,*] & *Leonie Klock* [a,b,c,*], *Johanna Bächle* [d], *Laura Stoll* [d], *Patrick Giemsa* [d], *Marie Fuchs* [d], *Nikola Schoofs* [d], *Christiane Montag* [d], *Jürgen Gallinat* [a], *Simone Kühn* [a,e,+]

[a] University Medical Center Hamburg-Eppendorf,
Clinic and Policlinic for Psychiatry and Psychotherapy,
Martinistraße 52, 20246, Hamburg, Germany

[b] Humboldt-Universität zu Berlin,
Berlin School of Mind and Brain,
Unter den Linden 6, 10099 Berlin, Germany

[c] Humboldt-Universität zu Berlin,
Department of Clinical Psychology,
Rudower Chaussee 18, 12489 Berlin, Germany

[d] Charité University Medicine and St. Hedwig-Krankenhaus,
Department of Psychiatry and Psychotherapy,
Große Hamburger Straße 5-11, 10115 Berlin, Germany

[e] Max Planck Institute for Human Development,
Lise-Meitner Group for Environmental Neuroscience,
Lentzeallee 94, 14195 Berlin

* indicates a shared first-authorship

[+] Correspondence to s.kuehn@uke.de

Supplementary Material

Methods

Participants

31 patients included in this study were receiving antipsychotic medication (amisulpride, *n*=12; risperidone, *n*=5; aripriprazole, *n*=3; quetiapine, *n*=4; clozapine, *n*=3; paliperidone, *n*=2; flupentixol, *n*=1; haloperidol, *n*=1; promethazine, *n*=1; pipamperon,*n*=2; fluphenazine, *n*=1; ziprasidone, *n*=1; chloproxiten n=1; flupenazyn n=1).

Results

Analysis controlling for the effects of medication. To assess whether medication influences the relationship between resting state connectivity and psychopathology, chlorpromazine-equivalents (CPZ) were calculated. CPZ did not correlate with functional connectivity in the precuneus given by spatial maps of the DMN (r(34) = -0.274, p = 0.117). A partial correlation between the ICA parameters (spatial maps) of the precuneus and the SANS composite score was still significant when controlling for the influence of CPZ (r(31) = -0.367, p = 0.036) as well as between the ICA parameter (spatial maps) of the precuneus and the SANS domain of apathy (r(31) = -0.513, p = 0.002) as well as with the multimodal apathy score (r(31) = -0.483, p = 0.004).

Analysis controlling for the effects of age, illness duration, and sex. A partial correlation controlling for the influence of age, illness duration, and sex was still significant between functional connectivity in the precuneus given by spatial maps of the DMN and the SANS composite score (r(29) = -0.472, p = 0.007), SANS domain of apathy (r(29) = -0.369, p = 0.041) and the multimodal apathy score (r(29) = -0.401, p = 0.025).

A partial correlation that additionally included CPZ as well as age, illness duration and sex yielded significant results for the correlation between the ICA parameters (spatial maps) of the precuneus and the SANS composite score (r(27) = -0.444, p = 0.016) as well as for the

multimodal apathy score (r(27) = -0.389, p = 0.037) but not for the SANS domain of apathy (r(27) = -0.356, p = 0.058).

Figure

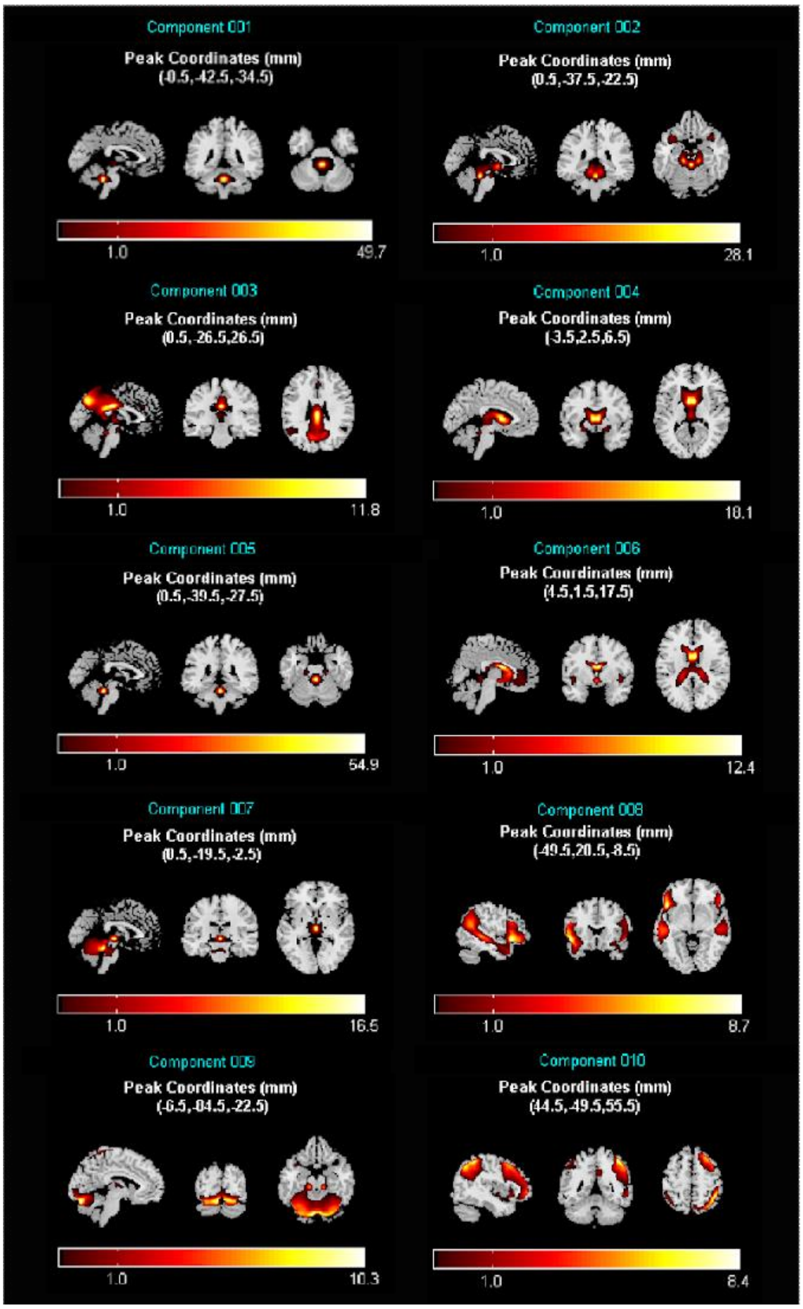

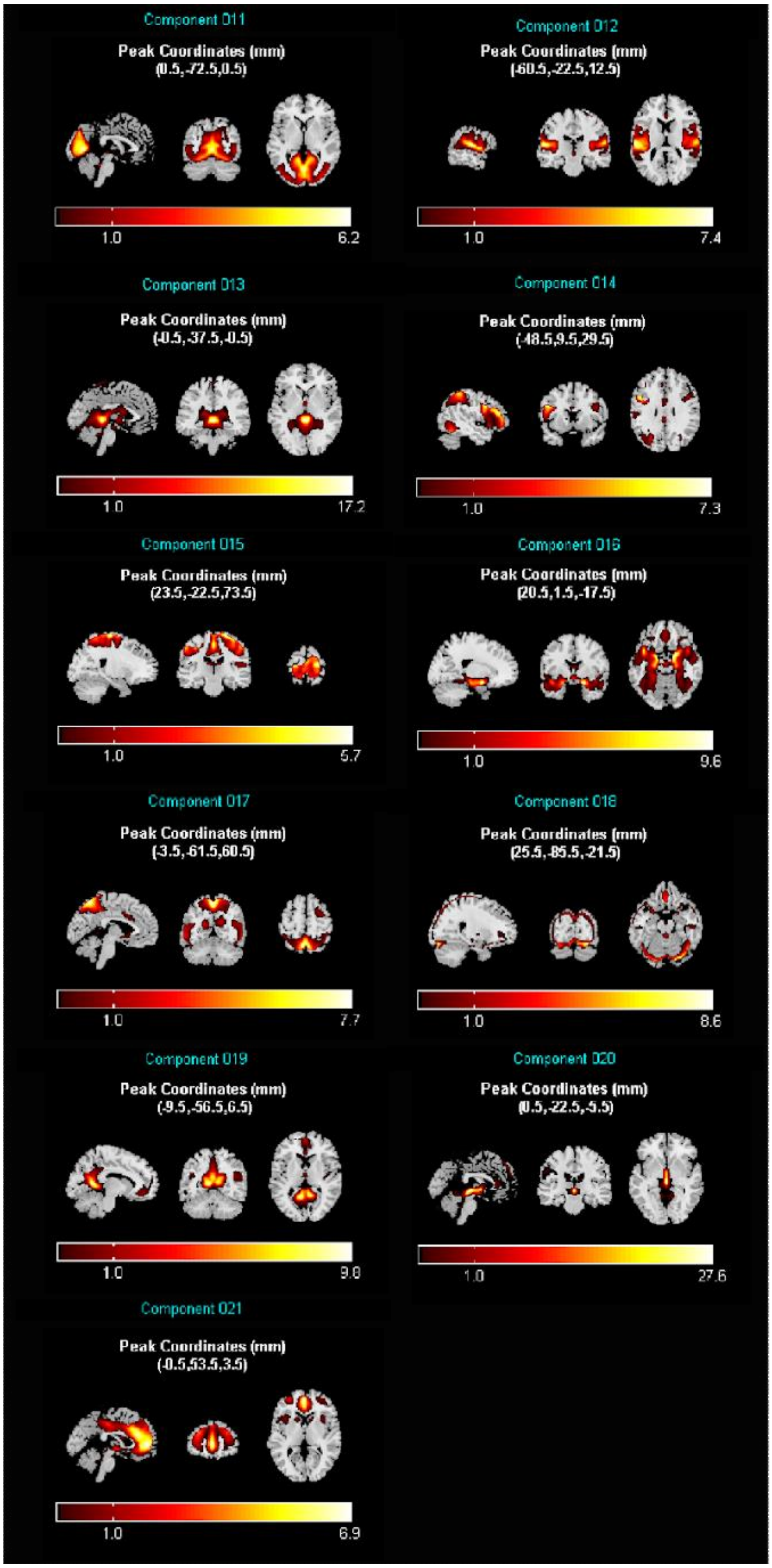

Figure S1. All components extracted by GIFT toolbox. The following components were considered: basal ganglia (component 4), salience (component 8), higher visual (component 9), executive control (component 10), primary visual (component 11), auditory (component 12), visuospatial (component 14) and default mode network (component 19).
